# Case of Strangulated Inguinal Hernia, in Which the Operation Was Performed a Second Time in the Same Place

**Published:** 1826-07

**Authors:** 

**Affiliations:** the Middlesex Infirmary, *Great Pulteney Street.*


*Case of Strangulated Inguinal Hernia, in which the Operation was performed a second time in the same place.* Treated by Mr. Boyle, at the Middlesex Infirmary, *Great Pulteney Street.*

On the morning of the 20th of November, 1825, Mr. Boyle was called to visit Thomas Rook, residing in Turner's-court, St. Martin's-lane, labouring under strangulated inguinal hernia. This patient had been operated on about four years before, and had worn a truss till within a short period of the present account, when it was left off in consequence of being out of repair. He stated that, at nine o'clock the preceding evening, (twelve hours previously to his being visited,) he suddenly felt a sensation as if something had given way in the groin. On examination, an unusual enlargement presented itself, which increased up to the time specified above, accompanied by gradually increasing pain over the whole abdominal region, great restlessness, and nausea.

The taxis was repeatedly tried in vain. Pounded ice was applied to the hernial tumor. An ounce of castor-oil was administered, but immediately returned; and a tobacco enema was ordered to be prepared.

At twelve o'clock, the pain and the disordered state of the stomach had increased; the hernial tumor had become enlarged, and the countenance was demonstrative of considerable suffering. The tobacco enema was now administered; but, being unattended with benefit, and there being no immediate means of procuring a warm bath, the gentlemen present (Mr. Jewel, Mr. Wade, and Mr. Boyle,) coincided in opinion that an operation should not be longer deferred.

This was accordingly commenced by the latter gentleman, who carried an incision from about three-fourths of an inch above the abdominal ring, in a parallel direction to the cicatrix of the former operation, to the bottom of the tumor: thus avoiding, as much as possible, the old indurated seam of the integuments. The skin, of necessity, was divided somewhat behind the centre of the tumor. The cellular membrane was now exposed, and was observed to be so condensed and thickened as to have almost changed its structure, requiring particularly cautious dissection, from the further circumstance of its being composed of five separate layers, like fascia. These having been divided, the hernial sac was perforated with due caution: it was laid open to the bottom of the tumor, and about a table-spoonful of serous fluid escaped. The stricture was now sought for, and was found at the inner margin of the tendon of the external oblique muscle, and was removed by a gently conducted sawing motion of a probepointed bistoury; the index finger of the left hand serving as a director. Every part of the protruded intestine had a livid appearance: it was returned as quickly, yet as gently, as possible, and the parts were brought in apposition; but, from their peculiarly thickened state, it was deemed necessary to unite them by two stitches, after which the parts were dressed in the ordinary manner, with adhesive straps, &c. All pain and uneasiness were removed on freeing the strangulated gut. The bowels were soon after opened by a dose of castor-oil; and, at the expiration of six days from the performance of the operation, the parts were completely united, and the patient was in perfect health.

